# Multi-Field Coupling- and Data-Driven-Based Optimization of Cooling Process Parameters for Planetary Rolling Rolls

**DOI:** 10.3390/ma18174111

**Published:** 2025-09-01

**Authors:** Fengli Yue, Yang Shao, Hongyun Sun, Jinsong Liu, Dayong Chen, Zhuo Sha

**Affiliations:** 1 College of Automotive and Transportation, Shenyang Ligong University, Shenyang 110159, China; 15588358153@163.com (Y.S.); sunhy5006@163.com (H.S.); zhuosha0820@163.com (Z.S.); 2 Shi Changxu Materials Innovation Center, Institute of Metal Research, Chinese Academy of Sciences, Shenyang 110016, China; jsliu@imr.ac.cn (J.L.); dychen15b@imr.ac.cn (D.C.); 3 College of Materials Science and Engineering, Shenyang Ligong University, Shenyang 110159, China

**Keywords:** three-roll planetary rolling, machine learning, numerical simulation, spray cooling, heat transfer

## Abstract

In the three-roll planetary rolling process, excessively high surface temperature of the rolls can easily lead to copper adhesion, deterioration of roll surface quality, shortened rolling lifespan, and severely affect the quality of copper tube products as well as production efficiency. To improve the cooling efficiency of the roll cooling system, this study developed a fluid–solid–heat coupled model and validated it experimentally to investigate the effects of nozzle diameter, spray angle, and axial position of the spray ring on the cooling performance of the roll surface. Given the low computational efficiency of finite element simulations, three machine learning models—Random Forest (RF), Gradient Boosting Decision Tree (GBDT), and Support Vector Machine (SVM)—were introduced and evaluated to identify the most suitable predictive model. Subsequently, the Particle Swarm Optimization (PSO) algorithm was employed to optimize the geometric parameters of the spray ring. The results show that the maximum deviation between the coupled model predictions and experimental data was 4.36%, meeting engineering accuracy requirements. Among the three machine learning models, the RF model demonstrated the best performance, achieving RMSE, MAE, and R^2^ values of 1.7336, 1.3203, and 0.9082, respectively, on the test set. The combined RF-PSO optimization approach increased the heat transfer coefficient by 44.72%, providing a robust theoretical foundation for practical process parameter optimization and precision tube manufacturing.

## 1. Introduction

**Figure 1:**
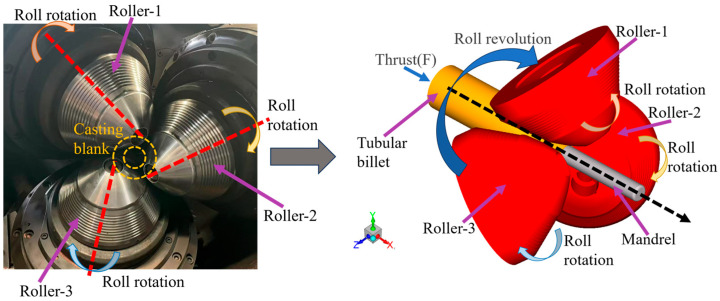
Schematic diagram of three-roll planetary rolling.

Three-roll planetary rolling, as a continuous metal forming process, is widely used in the manufacturing of high-end precision copper tubes. As illustrated in Figure 1, this processing method utilizes three sets of high-speed rotating rolls to achieve uniform diameter reduction and axial elongation of the metal billet. It not only demonstrates advantages such as high forming efficiency and minimal dimensional tolerances, but also significantly enhances the microstructure and mechanical properties of the final product [1,2]. Three-roll planetary rolling is a complex process involving the coupling of thermal, mechanical, and fluid fields. During this process, intense friction and the heat generated from the plastic deformation of the copper billet cause a rapid rise in temperature at the interface between the rolls and the billet [3,4,5]. Without proper cooling system control, localized overheating on the roll surface may occur, leading to copper adhesion [6]. Such thermal conditions not only cause severe surface temperature fluctuations but also accelerate thermal fatigue of the rolls, ultimately degrading their surface quality [7,8]. Garrd et al. [9] found that when the surface temperature of the rolls is within the range of 473 K to 523 K, the surface adhesion phenomenon of copper will be significantly enhanced. Meanwhile, Wang et al. [10] found that when the working temperature of 3Cr2W8V die steel exceeds 923 K, its failure process will accelerate significantly. Therefore, accurately understanding the temperature distribution and its variation on the roll surface, as well as optimizing the cooling efficiency of the roll cooling system, and maintaining the surface temperature of the rolls within a reasonable range are the keys to improving the quality of copper tubes and extending the service life of the rolls.

At present, roll cooling primarily relies on a spray ring structure, which atomizes and sprays cooling liquid onto the roll surface through multiple nozzles. Due to the complex structure of the rolling mill and the dynamic motion of its components, conducting direct experimental studies is challenging. As a result, most research on spray rings depends on finite element simulations or equivalent experimental methods. Fu et al. [11], Silk et al. [12], and Niu et al. [13] developed equivalent spray cooling experimental setups using flat plates as research subjects to investigate the effects of spray angle and flow rate on heat transfer efficiency and heat flux. Liu et al. found that the far-field spray cone angle decreases sharply with increasing pressure differential [14]. Yan et al., using Computational Fluid Dynamics (CFD) methods, discovered that surface heat flux initially increases and then decreases as the spray distance grows [15]. Sheikh et al. [16], Speicher et al. [17], and Koohbor et al. [18] employed the finite element method to develop temperature field models for flat plate rolling, enabling accurate prediction of temperature distributions. Chen et al. proposed a Peaceman–Rachford (PR) scheme model and found that roll displacement can suppress edge thermal expansion [19]. Azene et al. [20] and Apostor et al. [21] demonstrated that increasing the cooling liquid flow rate can enhance the cooling capacity of roll surfaces. However, due to the unique spatial configuration of the three-roll planetary rolling system, the influence mechanisms of spray ring parameters on cooling performance are more complex. Therefore, conducting optimization studies on spray ring parameters specifically for this specialized structure not only addresses the limitations of existing research but also provides a direct theoretical basis for setting process parameters in practical production.

Although the finite element method can accurately reflect heat exchange behavior and produce precise results, it heavily relies on boundary conditions, involves high computational costs, and suffers from low efficiency in multi-parameter scenarios, making it difficult to respond quickly to parameter changes. With the advancement of data-driven approaches, machine learning has shown great potential in multi-factor modeling and temperature field prediction due to its superior nonlinear fitting capabilities and high prediction efficiency. Rahaman et al. [22] and Thiercelin et al. [23] constructed various machine learning models to achieve high-precision prediction of the phase transformation temperature of different metallic materials. Yue et al. demonstrated that for TP2 copper tube wall thickness prediction, the Support Vector Machine (SVM) model outperformed BP, RBF, and RF models in terms of accuracy and fitting quality [24]. Deshannavar et al. compared the performance of RF and SVM in predicting the heat transfer coefficient of spray cooling, with results showing that SVM achieved higher accuracy [25]. Wu et al. employed small-sample machine learning techniques and found that even with a 50% reduction in data volume, prediction accuracy improved by 3.5% [26]. These studies indicate that machine learning holds great potential for temperature prediction in metal forming processes. However, it has not yet been applied to the prediction of surface temperature fields in three-roll planetary rolls. Therefore, the rational selection and application of machine learning models can not only improve the efficiency of parameter optimization but also lay a solid foundation for the development of intelligent manufacturing platforms for copper tube processing.

This study addresses the issue of insufficient cooling regulation in three-roll planetary mills, where the roll surface is prone to excessive temperatures during production. A fluid–solid–heat coupled finite element model of the planetary rolls is established to analyze the influence mechanisms of nozzle diameter, spray angle, and axial arrangement on the surface temperature distribution of the rolls. On this basis, multiple machine learning algorithms are introduced to predict the temperature field in critical regions. Through comprehensive comparison of prediction accuracy and generalization capability among different models, the optimal predictive algorithm is identified, enabling the development of a rapid prediction model for roll surface temperature fields. Furthermore, the Particle Swarm Optimization (PSO) algorithm is employed to optimize the geometric parameters of the spray ring, achieving optimal cooling performance and providing a theoretical foundation for the optimization and improvement of the cooling system in three-roll planetary mills.

## 2. Multiphysics Coupling Model

The roll cooling process involves the flow of coolant inside the spray ring and the interaction between droplets and the roll surface. In this study, a mathematical model is developed, where the fluid inside the spray ring and the spray environment are modeled as a continuous phase, while the droplets are simulated using a Discrete Phase Model (DPM).

### 2.1. Control Equation

The computational analysis based on multiphysics coupling requires the fluid domain to satisfy the continuity equation, momentum conservation equation, and energy conservation equation [27,28]. Additionally, the interaction between the discrete phase (droplets) and continuous phase in the DPM must be considered [29].

(1) Continuity equation

(1) $$\frac{\partial \rho_{f}}{\partial t}+\nabla \cdot \left(\rho_{f}u_{f}\right)=0$$

where $\rho_{f}$ is continuous phase density, $u_{f}$ is continuous phase velocity, and t is time.

(2) Momentum conservation equation

(2) $$\left\{\begin{array}{l} \frac{\partial \left(\rho_{f}u_{f,x}\right)}{\partial t}+\nabla \cdot \left(\rho_{f}u_{f,x}U\right)=\nabla \cdot \left(\eta \nabla u_{f,x}\right)-\frac{\partial p}{\partial x}+F_{x}\\ \frac{\partial \left(\rho_{f}u_{f,y}\right)}{\partial t}+\nabla \cdot \left(\rho_{f}u_{f,y}U\right)=\nabla \cdot \left(\eta \nabla u_{f,y}\right)-\frac{\partial p}{\partial y}+F_{y}\\ \frac{\partial \left(\rho_{f}u_{f,z}\right)}{\partial t}+\nabla \cdot \left(\rho_{f}u_{f,z}U\right)=\nabla \cdot \left(\eta \nabla u_{f,z}\right)-\frac{\partial p}{\partial z}+F_{z} \end{array}\right.$$

where U is the velocity vector, η is the dynamic viscosity, and $F_{x},\;F_{y},\;F_{z}$ is the component of the external force term in all directions.

(3) Energy conservation equation

(3) $$\frac{\partial \left(\rho_{f}E_{f}\right)}{\partial t}+\nabla \cdot \left(\rho_{f}u_{f}E_{f}\right)=\nabla \cdot \left(k_{f}\nabla T_{f}\right)+\nabla \cdot \left(\tau \cdot u_{f}\right)+S_{DPM}$$

where $E_{f}$ is the total energy of the fluid, $k_{f}$ is the thermal conductivity of the fluid, $T_{f}$ is the temperature of the fluid, and $S_{DPM}$ is the energy exchange between the discrete and continuous phases.

(4) Turbulence model

(4) $$\frac{\partial (\rho k)}{\partial t}+\nabla \cdot (\rho k\vec{v})=\nabla \cdot \left[\left(\mu +\frac{\mu_{t}}{\sigma_{k}}\right)\nabla k\right]+G_{k}-\rho \varepsilon$$

(5) $$\frac{\partial (\rho \varepsilon )}{\partial t}+\nabla \cdot (\rho \varepsilon \vec{v})=\nabla \cdot \left[\left(\mu +\frac{\mu_{t}}{\sigma_{\varepsilon }}\right)\nabla \varepsilon \right]+C_{1}f_{1}\frac{\varepsilon }{k}G_{k}-C_{2}f_{2}\rho \frac{\varepsilon^{2}}{k}$$

where the constant $C_{1}$ is 1.44 and $C_{2}$ is 1.92, which is the turbulence generation term.

(5) DPM model

When simulated using DPM model, the behavior of droplets is mainly described by the equations of motion, energy, and mass:

(6) $$\left\{\begin{array}{l} \frac{du_{p,x}}{dt}=F_{D}\left(u_{f,x}-u_{p,x}\right)+\frac{g_{x}\left(\rho_{p}-\rho_{f}\right)}{\rho_{p}}-\frac{1}{\rho_{p}}\frac{\partial p}{\partial x}\\ \frac{du_{p,y}}{dt}=F_{D}\left(u_{f,y}-u_{p,y}\right)+\frac{g_{y}\left(\rho_{p}-\rho_{f}\right)}{\rho_{p}}-\frac{1}{\rho_{p}}\frac{\partial p}{\partial y}\\ \frac{du_{p,z}}{dt}=F_{D}\left(u_{f,z}-u_{p,z}\right)+\frac{g_{z}\left(\rho_{p}-\rho_{f}\right)}{\rho_{p}}-\frac{1}{\rho_{p}}\frac{\partial p}{\partial z} \end{array}\right.$$

where $u_{p}$ is the particle velocity vector, $\rho_{p}$ is the particle density, and $F_{D}$ is the drag acceleration coefficient per unit mass.

The solid region is the cooled part, so the governing equation is mainly used to describe the heat conduction of the roll, which is mainly determined by the heat conduction equation and the heat transfer between the droplet and the roll surface:

(7) $$\rho_{s}c_{p}\frac{\partial T_{s}}{\partial t}=\nabla \cdot \left(k_{s}\nabla T_{s}\right)$$

where $\rho_{s}$ is roll density, $c_{p}$ is roll specific heat capacity, $T_{s}$ is roll temperature, and $k_{s}$ is roll thermal conductivity.

### 2.2. Fluid-Solid-Heat Coupled Model

Spray ring and fluid-solid-heat coupling model are constructed, respectively, by using the Fluent program. Firstly, the parameters of spray ring are calculated, and then the results of velocity and flow rate are transmitted to fluid-solid-heat coupling simulation as part of input conditions.

**Figure 2:**
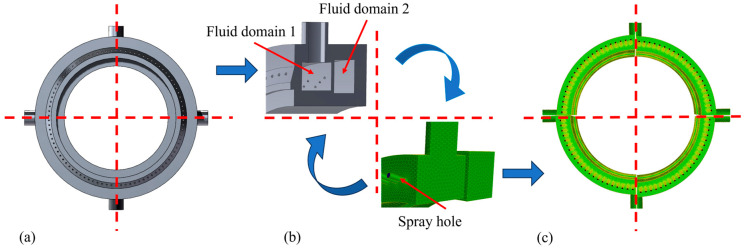
(**a**) Spray ring model, (**b**) mesh drawing, and (**c**) mesh model of spray ring.

The spray ring was modeled as shown in Figure 2a,b. As illustrated in Figure 2b, the interior of the spray ring consists of two annular fluid domains, which are interconnected by several orifices with a diameter of 2 mm. The meshing was performed using the Meshing tool in the Fluent module. The internal fluid domains of the spray ring were first extracted and then discretized using polyhedral elements. Given the small dimensions of the spray and connecting holes, local mesh refinement was applied to the hole profiles and internal connecting regions to ensure high computational accuracy. The final meshed internal fluid domains are presented in Figure 2c.

**Figure 3:**
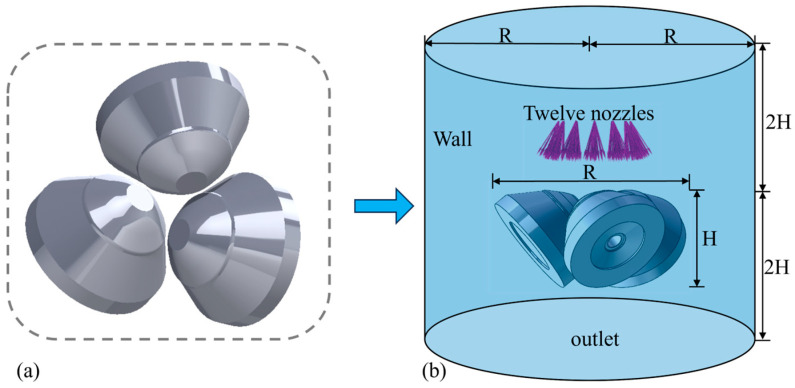
(**a**) Planetary roller model, and (**b**) fluid–solid–thermal coupling model.

According to the roll drawing and the spatial position of the field roll, the geometric model, as shown in Figure 3a, is established. In terms of mesh division, considering that the roll is a key calculation area, the area is densified, 400 nodes are evenly distributed on the roll surface along the circumferential direction, and hexahedral elements are used for mesh drawing. The fluid domain is a large cylinder wrapping the roll model, and the specific size is shown in Figure 3b.

### 2.3. Parameter Setting

**Figure 4:**
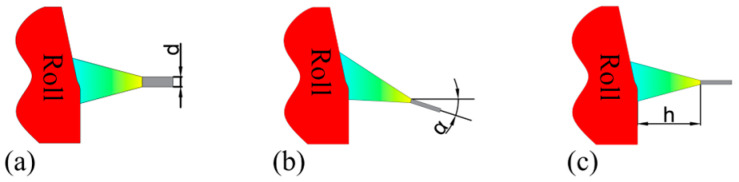
Simulation parameters: (**a**) blowhole diameter, (**b**) spray angle, and (**c**) axial position.

Three key parameters—diameter of water spray hole (d), angle of water spray (*α*), and axial position of water spray ring (h)—are selected as the research objects, as shown in Figure 4, to systematically explore their influence on the size and distribution of temperature field on roll surface. It is important to consider that in an actual rolling mill production environment, roll installation is affected by multiple factors such as pipe size, production process requirements, and rolling mill space constraints, and the size and axial movement range of water spray ring are also strictly limited. Five gradient levels are set for each parameter, and the specific parameters are shown in Table 1.

In the numerical simulation, the Discrete Phase Model (DPM) is employed to quantitatively analyze the cooling effectiveness of the rolls under varying structural parameters and installation positions of the spray ring. The rolls are fabricated from 3Cr2W8V alloy steel, and the cooling medium was a mixture of water and emulsion. The addition of the emulsion alters the thermophysical properties of the coolant and forms an oil film on the roll surface, thereby affecting the cooling performance [30]. In this study, the emulsion concentration was set to 5%, with Table 2 listing the material’s thermophysical properties and simulation parameters that remained constant throughout the analysis [31].

### 2.4. Boundary Conditions

#### 2.4.1. Inlet and Outlet

The spray ring model adopts pressure boundary conditions for both inlet and outlet, with its computational results and structural parameters serving as the inlet boundary conditions for the fluid-solid-heat coupling model. In this coupled model, cone nozzles are employed for the spray configuration. Considering computational costs and experimental constraints, this study reduces the number of nozzles while maintaining consistent spray coverage through extensive experimental validation, ultimately determining an optimal configuration of 12 nozzles.

#### 2.4.2. Roller

During the rolling process, the temperature rise of the rolls is primarily influenced by heat conduction from the copper tube and frictional heating. Heat exchange through radiation with the surrounding environment is relatively minor and thus neglected in this study to simplify the model. The analysis focuses principally on convective heat transfer with the coolant.

(8) $$-{\left.k\frac{\partial T}{\partial n}\right|}_{\Gamma }=q_{\mathrm{conv}}+q_{\mathrm{sens}}+q_{\mathrm{evap}}$$

In the formula, $q_{\mathrm{conv}}$ mainly considers convective heat transfer between liquid droplets and roll surface, $q_{\mathrm{sens}}$ mainly considers temperature rise during coolant heat exchange, and $q_{\mathrm{evaq}}$ mainly considers evaporation latent heat absorption during coolant heat exchange [32]:

(9) $$\left\{\begin{array}{l} q_{\mathrm{conv}\;}=h\left(T_{s\;}-T_{\mathrm{liq}\;}\right)\\ q_{\mathrm{sens}\;}={\dot{m}}_{\mathrm{evap}\;}c_{p}\left(T_{\mathrm{boil}\;}-T_{\mathrm{liq}\;}\right)\\ q_{\mathrm{evap}\;}={\dot{m}}_{\mathrm{evap}\;}L \end{array}\right.$$

where $T_{s}$ is the roll temperature; $T_{\mathrm{liq}}$ is the initial temperature of cooling liquid, $T_{\mathrm{boil}}$ is the boiling temperature of cooling liquid, usually 373.15 K; and L is the latent heat of vaporization of cooling liquid.

Considering the model size limitation and computational complexity, the roll surface is set to wall-film boundary conditions, without considering the secondary cooling effect caused by the rebound of droplets after collision. This treatment method is more suitable for the actual cooling behavior, and it is helpful to simplify and load the boundary conditions [33].

#### 2.4.3. Evaporation Boundary

During the cooling process of the roll, the cooling liquid at low temperature and the roll surface at high temperature will partially evaporate at the moment of contact. In this process, not only sensible heat transfer of liquid phase exists, but also obvious latent heat exchange exists. In order to increase the simulation accuracy of multiphase coupling, the evaporation mass rate in Equation (10) is mainly obtained from the formula [34]

(10) $${\dot{m}}_{\mathrm{evap}\;}=k_{c}\cdot A_{m}\cdot \rho_{l}\cdot \left(Y_{\mathrm{sat}\;}-Y_{\infty }\right)$$

where $k_{c}$ is the mass transfer coefficient, which is related to Reynolds number and Schmidt number; $A_{m}$ is evaporation area; and $Y_{\mathrm{sat}}$ is saturation mass fraction.

#### 2.4.4. Liquid Film Flow

During the spray cooling process on the roll surface, not all cooling liquid completely evaporates. A portion of the liquid fails to fully vaporize due to insufficient heat absorption, excessive flow velocity, vapor barrier effects, or inadequate surface contact, and ultimately drains away in liquid form.

(11) $$u_{f}=\frac{\rho \cdot g\cdot \delta^{2}}{3\cdot \mu }$$

(12) $$\frac{d}{dt}(\rho \cdot \delta )+\nabla \cdot \left(\rho \cdot \delta \cdot {\vec{u}}_{f}\right)={\dot{m}}_{\mathrm{in}}-{\dot{m}}_{\mathrm{evap}}$$

Here, δ is the thickness of the liquid film on the roll surface and μ is the viscosity of the liquid.

## 3. Simulation Results and Experimental Verification

### 3.1. Simulation Results of Surface Temperature Field

**Figure 5:**
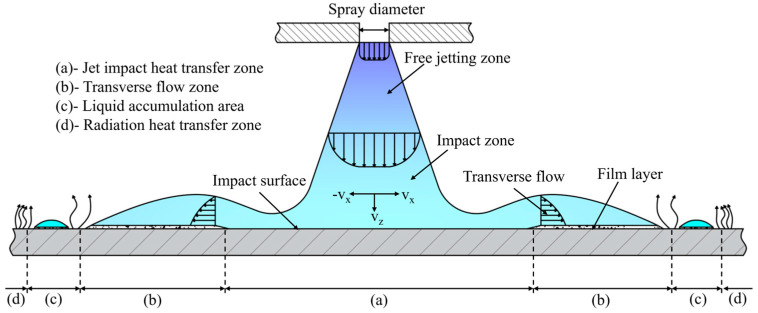
Heat transfer zone on roll surface during cooling.

Figure 5 shows the cooling process of the roll surface by the spray ring. It can be seen from the figure that after the cooling liquid is sprayed from the spray ring, it first directly impinges on the roll surface, forming the jet impact heat transfer zone with the most significant cooling effect [11]. In this zone, the cooling liquid impinges on the surface at high speed and quickly removes surface heat. Subsequently, the cooling liquid flows along the roll surface, gradually forming a liquid film on the surface, and heat is removed by convective heat transfer with the liquid film, as depicted in Figure 5b. With the further movement of liquid, influenced by surface tension and boundary conditions, the cooling liquid accumulates locally in the peripheral area, forming a liquid dam, shown in Figure 5c, which is a small liquid accumulation area, and its fluidity weakens and the heat transfer effect decreases. At the outermost part of the area, the surface is basically covered by liquid, and the heat mainly dissipates outward through radiation, resulting in the weakest cooling efficiency.

**Figure 6:**
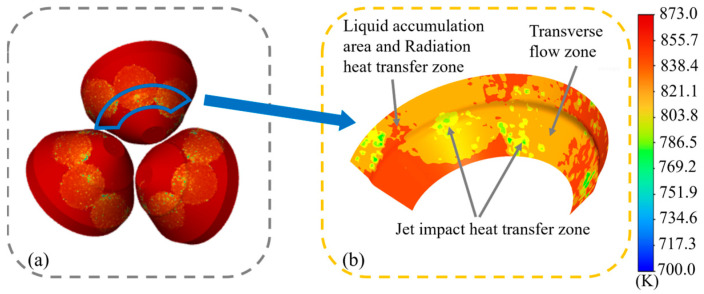
(**a**) Roll temperature field and (**b**) surface temperature field.

Considering factors such as simulation time and cost, a fixed time step was adopted for the simulation. The analysis was conducted over 2000 steps to evaluate the surface temperature values and distributions of the rolls under various spray ring configurations. The simulation results are presented in Figure 6a. It can be observed that the surface temperature distributions of the three rolls exhibit a high degree of similarity. Therefore, a single roll was selected as the subject for subsequent analysis. Figure 6b shows the temperature contour map of the selected section. It is evident that the temperature contour on the roll surface demonstrates a pattern consistent with that shown in Figure 5a–d. The lowest temperatures appear in the jet impact heat transfer zone, followed by the transverse flow zone, while relatively higher temperatures are observed in the zones with liquid accumulation and radiation heat transfer zone. However, due to the geometric shape of the roll surface and the installation angle, the spatial distribution characteristics of the transverse flow zone and the jet impact heat transfer zone vary significantly. In the central region of the roll surface, the two zones are approximately concentric, while towards the sides of the roll, where the surface curvature increases, a notable spatial offset between the jet impact heat transfer zone and the transverse flow zone becomes apparent.

### 3.2. Experimental Verification

**Figure 7:**
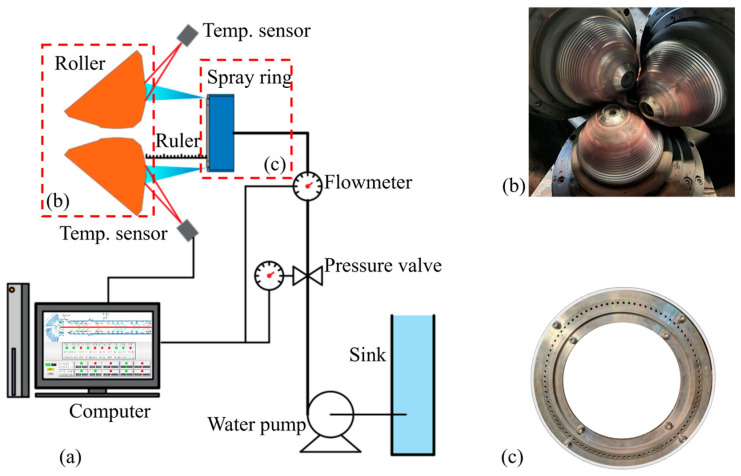
(**a**) Experimental setup scheme, (**b**) roller structure, (**c**) water spray ring structure.

The experimental setup, as illustrated in Figure 7, consists of four main components: a coolant supply system, a high-temperature roll, a spray ring, and an infrared temperature measurement system. Cooling liquid was used as the working fluid in the experiments, operating under constant pressure and ambient temperature conditions (approximately 300 K). The roll surface was heated using actual production processes, as shown in Figure 7b, to simulate the high-temperature condition of the roll under real operating scenarios. The spray ring, fabricated from aluminum, as shown in Figure 7c, allows for the adjustment of experimental parameters by varying its axial position or replacing the end face. The temperature measurement system employed a high-precision infrared pyrometer with a spectral range of 0.7–2.6 µm. The device featured an adjustable emissivity range from 5% to 120% and offered a measurement accuracy better than 0.5%.

Using the experimental setup shown in Figure 7, a series of tests were carried out by varying the axial position of the spray ring (with a nozzle diameter of 2.5 mm and a spray angle of 18°), and the surface temperature values of the rolls after cooling for 10 s were recorded, respectively. To quantitatively evaluate the cooling performance, 300 feature points were uniformly selected within the study area for temperature data collection and statistical analysis. The resulting average surface temperatures are presented in Table 3. It can be seen that due to the large number of model grids, the simulation time has been significantly reduced, resulting in a large difference between the simulation results and the experimental results. However, the overall change trend is consistent; that is, as the axial distance increases, the average surface temperature of the rolls initially decreases and then increases. This trend is primarily due to the fact that, as the spray distance increases, the coverage area expands, significantly improving the uniformity of cooling. However, when the distance becomes too large, the impingement intensity of the cooling liquid flow weakens, and part of the coolant fails to effectively reach the measurement area, leading to reduced cooling efficiency.

The heat transfer coefficient is a key parameter characterizing the efficiency of heat exchange. In this study, Formula (13) is used to calculate the heat transfer coefficient under each parameter combination:

(13) $$h=-\frac{\rho cV}{At}\cdot \ln\left(\frac{T(t)-T_{\infty }}{T_{i}-T_{\infty }}\right)$$

where $T_{i}$ is the initial roller surface temperature, $T\left(t\right)$ is the average roller temperature after cooling, $T_{\infty }$ is the cooling liquid temperature, ρ is the density of the material, c is the specific heat capacity at constant pressure of the material, V is the volume of the study area, A is the surface area of the study area, and t is the cooling time.

**Figure 8:**
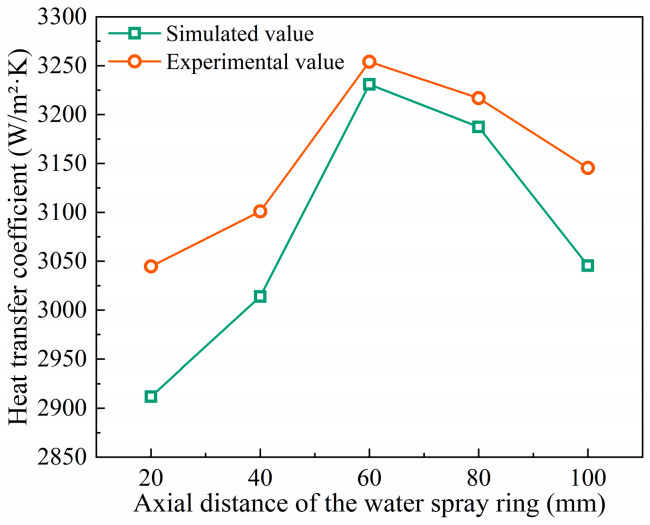
Comparison of experimental and simulated heat transfer coefficients.

Based on the experimental measurements and numerical simulations under identical parameter conditions, the average surface temperatures were obtained and used to calculate the corresponding heat transfer coefficients. The results are shown in Figure 8. It can be seen that the maximum deviation between the simulation and experimental results is 4.36%, while the minimum deviation is 0.92%. During the numerical simulation, the flow rate and velocity of cooling liquid from each individual nozzle were kept consistent with the experimental conditions. As a result, the local cooling behavior exhibits quantifiable consistency between simulation and experiment, effectively capturing the local cooling characteristics observed in the experimental data.

## 4. Simulation Results and Analysis

### 4.1. The Influence of Flow Velocity and Flow Rate

**Figure 9:**
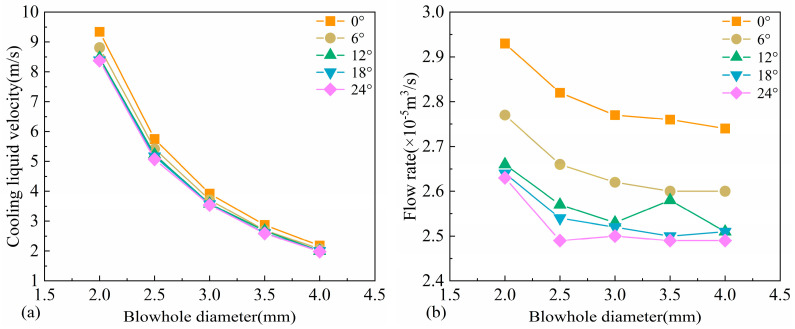
(**a**) Velocity and (**b**) flow rate of cooling liquid spout under various parameters.

Due to the boundary layer effects along the inner wall of the spray nozzle, the coolant velocity in the near-wall region is significantly reduced. To eliminate local velocity discrepancies, the velocity within the nozzle was arithmetically averaged, and the results are shown in Figure 9a. The data indicate that, under the same pressure conditions, the cooling liquid velocity decreases significantly with increasing nozzle diameter. Additionally, as the spray angle increases, the cooling liquid velocity gradually decreases; however, the velocity variation becomes less pronounced at larger spray angles. This phenomenon can be attributed to two primary factors: first, with a larger nozzle diameter, the pressure energy converted into kinetic energy per unit area is reduced; second, an increased spray angle extends the flow path of the coolant and leads to greater energy dissipation. The corresponding coolant flow rates for different parameter combinations are presented in Figure 9b. The overall trend of the flow rate shows a similar decreasing pattern to that of the velocity. However, when the nozzle diameter exceeds 3 mm, the reduction in flow rate becomes more gradual. According to the continuity equation and the schematic shown in Figure 9a, the reduction in flow velocity is substantially greater than the compensating effect caused by the increase in cross-sectional area. Moreover, with increasing spray angle, the coolant flow rate exhibits a notable decline, indicating that the spray angle has a significant influence on the coolant delivery rate.

### 4.2. The Influence of Average Temperature

**Figure 10:**
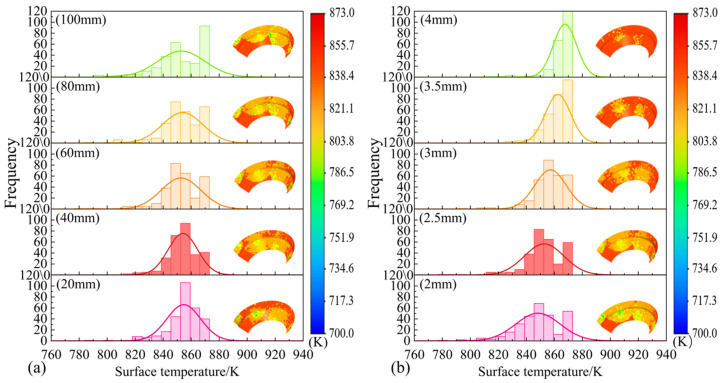

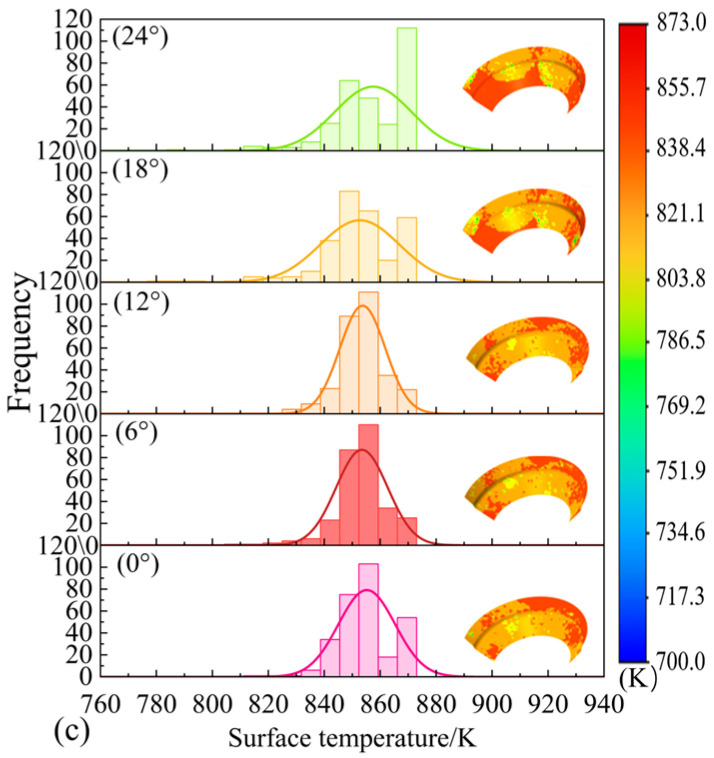
Surface temperature distribution of the roll under different (**a**) axial distances, (**b**) diameters, and (**c**) spray angles.

Figure 10 presents the temperature contour maps of the roll surface and the temperature distribution of characteristic points under various parameter combinations. As shown in Figure 10a, with increasing spray distance, the temperature histogram gradually broadens. At shorter spray distances, the cooling intensity is concentrated in the jet impact heat transfer zone, resulting in insufficient cooling at the edges and forming a pronounced temperature gradient. As the spray distance increases, the spray coverage area expands, significantly improving the uniformity of cooling.

Figure 10b clearly shows that the nozzle diameter has a significant impact on the roll surface temperature field. As the diameter increases, the temperature center gradually shifts toward the high-temperature region. With smaller nozzle diameters, a well-defined cooling zone forms on the roll surface. However, as the nozzle diameter increases, the contour of this cooling zone becomes less distinct and shrinks. When the diameter reaches 4 mm, it becomes difficult to form an effective cooling region on the roll surface. This phenomenon can be attributed to the fact that an increased nozzle diameter significantly reduces the coolant velocity and kinetic energy, thereby decreasing the heat removal caused by cooling liquid impingement. Additionally, larger nozzle diameters cause the cooling liquid to spread into a wider and thinner liquid film on the surface. As the liquid film evaporates, the area of the cooling zone on the roll surface gradually diminishes.

As shown in Figure 10c, with increasing nozzle spray angle, the cooling region in the temperature contour maps exhibits an upward shift, indicating a distinct spatial offset. At smaller spray angles, the jet impact heat transfer zone and the transverse flow zone remain relatively concentric, resulting in more symmetrical and uniform cooling. However, when the spray angle increases to the range of 18–24°, a noticeable misalignment appears between the jet impact heat transfer zone and the transverse flow zone, leading to reduced cooling uniformity. This offset is primarily caused by the outward displacement of the coolant contact position on the roll surface due to the change in nozzle angle.

**Figure 11:**
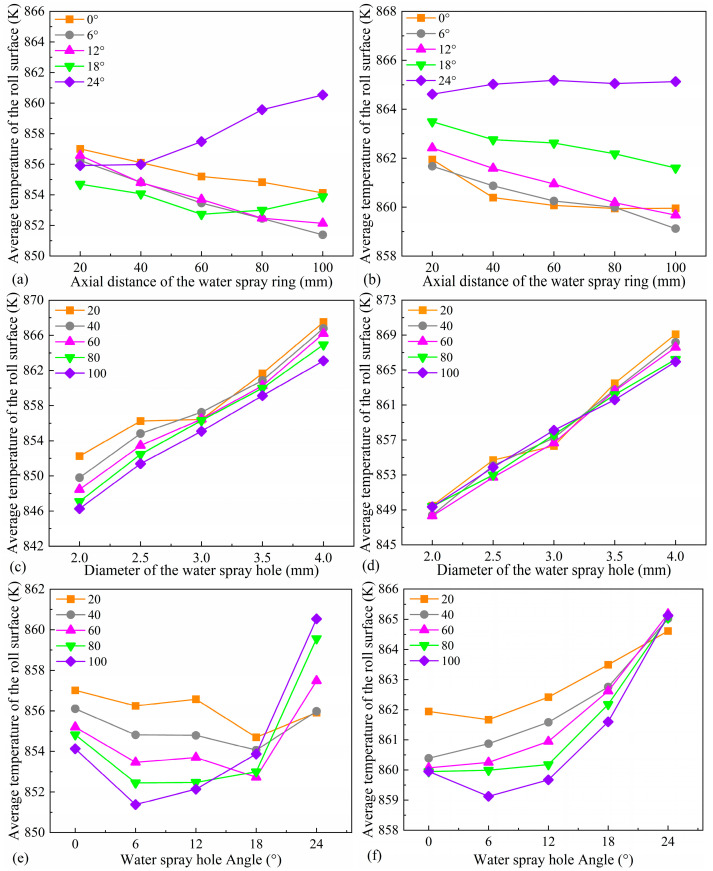
The variation of average temperature with distance (**a**) when d = 2.5 mm and (**b**) when d = 3.5 mm, the variation of average temperature with diameter (**c**) when *α* = 6° and (**d**) when *α* = 18°, the variation of average temperature with angle (**e**) when d = 2.5 mm and (**f**) when d = 3.5 mm.

Figure 11 illustrates the influence of spray ring geometric parameters on the average surface temperature of the roll. As shown in Figure 11a,b, under conditions of small spray angles and small nozzle diameters, the average surface temperature of the roll gradually decreases with increasing axial distance of the spray ring. This trend can be attributed to the expanded cooling coverage and improved spray quality resulting from the increased axial position, which enhances cooling uniformity and leads to lower surface temperatures [35,36]. However, at larger spray angles, the spray direction deviates from the target region, causing part of the coolant to impact areas outside the region of interest, resulting in an unexpected increase in average surface temperature with increasing axial distance.

As shown in Figure 11c,d, the average surface temperature of the roll increases significantly with further enlargement of the nozzle diameter. Although a larger nozzle diameter expands the coverage area of the coolant, a comprehensive analysis of Figure 9 reveals that both the flow velocity and flow rate of the coolant are substantially lower for larger nozzles compared to smaller ones. This results in weakened impingement intensity and reduced cooling effectiveness on the roll surface.

Compared with the effects of the spray ring’s axial position and nozzle diameter, the influence of nozzle angle on the average surface temperature of the roll exhibits a fluctuating trend, as shown in Figure 11e,f. Overall, the average temperature first decreases and then increases with increasing spray angle. Considering the coupling effect between nozzle diameter and axial position, it can be observed that the turning point in temperature variation shifts forward with larger spray angles. This phenomenon may result from the dual regulatory effect of nozzle angle on cooling conditions. On the one hand, it alters the position of the cooling region—as illustrated in Figure 10c, the cooling zone gradually shifts upward with increasing angle. On the other hand, as shown in Figure 9, variations in spray angle significantly modify the coolant velocity field, thereby affecting heat transfer efficiency. These combined effects lead to the observed fluctuations in average temperature. Studies indicate that, under fixed nozzle diameter and axial position, the spray angle has a pronounced impact on the uniformity of the surface temperature distribution of the roll.

### 4.3. The Influence on the Heat Transfer Coefficient of the Roll Surface

**Figure 12:**
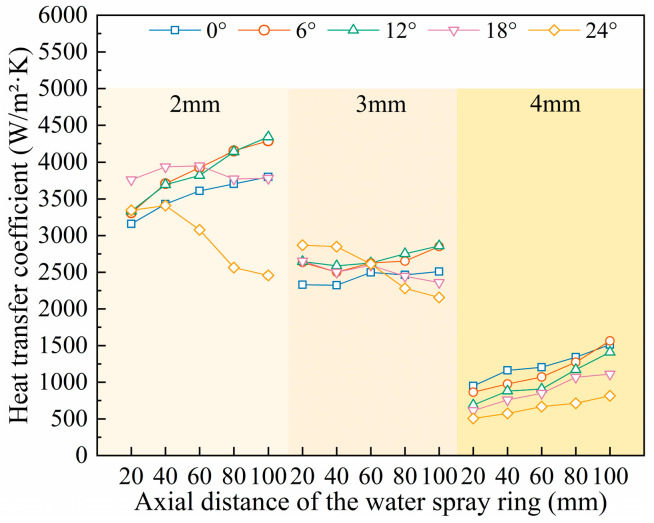
Change diagram of heat transfer coefficient under various parameters.

The geometric parameters of the spray ring, along with the coolant velocity, flow rate, and the latent heat of evaporation, have a significant influence on the distribution of the temperature field on the roll surface. Figure 12 illustrates the variation of the heat transfer coefficient with changes in axial position, nozzle diameter, and spray angle. The results show that nozzle diameter has a pronounced effect on the heat transfer coefficient. In particular, small-diameter nozzles exhibit generally higher heat transfer coefficients, while the coefficient tends to decrease with increasing diameter. For large-diameter nozzles, the heat transfer coefficient increases monotonically with decreasing spray angle and increasing axial distance. In contrast, the variation trend for small-diameter nozzles is more complex: under small spray angles, the heat transfer coefficient increases with axial distance, whereas under large spray angles, it shows the opposite behavior [37,38].

### 4.4. The Influence on the Equivalent Heat Flux on the Surface of the Rolls

**Figure 13:**
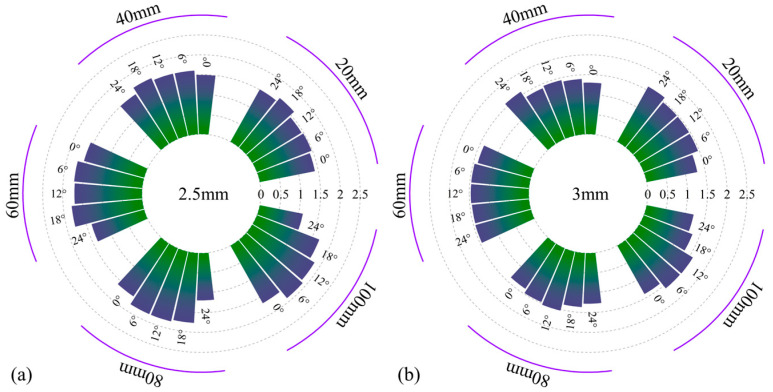
Equivalent heat flux (MW/m^2^) for (**a**) 2.5 mm and (**b**) 3 mm diameters.

Figure 13 presents the variation of the equivalent heat flux for small-diameter nozzles under different parameter conditions. It is clearly observed that the equivalent heat flux increases first and then decreases with increasing spray angle, and this trend becomes more pronounced with greater axial distance [11]. When the spray angle is 6° and the axial position is 100 mm, the equivalent heat flux reaches its peak value of 1.9 MW/m^2^. As the spray angle increases from 0° to 18°, the impingement effect of the coolant is enhanced, effectively disrupting the vapor film formed on the roll surface. However, when the angle increases from 18° to 24°, due to the conical geometry of the roll and the presence of a certain deflection and inclination angle during installation, the contact angle between the coolant and the surface becomes larger. This reduces the impingement intensity and weakens the ability of the coolant to break the vapor film, resulting in a decline in cooling performance.

## 5. Machine Learning Model

Although traditional finite element method has advantages in physical mechanism modeling, it has obvious limitations in engineering application. To overcome the limitations of complex modeling and high requirements for grid quality, this study introduces multiple machine learning models. Among them are the RF model, GBDT model, and SVM model. The concrete implementation scheme is that 125 groups of data obtained by finite element calculation under each parameter combination of water spray ring are sorted out, the training set and test set are divided to 80% and 20%, respectively, and then a machine learning model is constructed to realize rapid prediction of roll surface temperature field.

### 5.1. RF Model

RF is a machine learning algorithm for ensemble learning, which is widely applicable to regression tasks and has the characteristics of resisting overfitting and adapting to high-dimensional data [39,40]. The RF model has become one of the effective tools for solving nonlinear problems, and it makes a final prediction by integrating the prediction results of multiple decision trees:

(14) $$\begin{array}{l} \hat{y}=\frac{1}{T}\sum_{t=1}^{T}h_{t}(x)\\ h_{t}=\mathrm{BuildTree}\left(D^{\left(t\right)},D\right)\\ D^{\left(t\right)}\sim \mathrm{Bootstrap}(D,\left\lfloor r\cdot m\right\rfloor ) \end{array}$$

where T is the number of decision trees, D is the maximum depth of decision trees, and r is the sample proportion.

**Figure 14:**
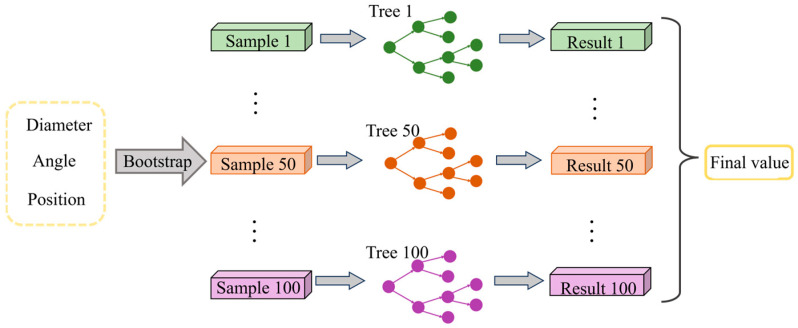
RF moder structure diagram.

The relationship between input and output parameters exhibits a complex nonlinear nature. To ensure that the model converges to an optimal solution while avoiding overfitting, multiple rounds of tuning were conducted. As a result, the number of decision trees in the model was set to 100. To balance the model’s learning capacity and computational efficiency, the maximum depth of each tree was limited to 5, and 80% of the training samples were randomly selected for each tree during training. This model structure effectively captures the complex mapping between input and output variables while maintaining a reasonable computational cost. The architecture is illustrated in Figure 14.

### 5.2. GBDT Model

GBDT is an iterative decision tree algorithm based on the additive model and distributed optimization idea. The core of this algorithm is to construct multiple weak learners (usually shallow decision trees) step by step and perform multiple iterations on the results. Each iteration trains a new base learner to fit the residual of the current model, so that it gradually approaches the target value [41,42]. The GBDT model is also based on the Boosting algorithm, and a new decision tree is established in the gradient direction of residual reduction:

(15) $$F_{k}(x)=\sum_{k=1}^{K}T\left(x;\varphi_{k}\right)$$

where $\varphi_{k}$ is the decision tree parameter and k is the number of decision trees.

**Figure 15:**
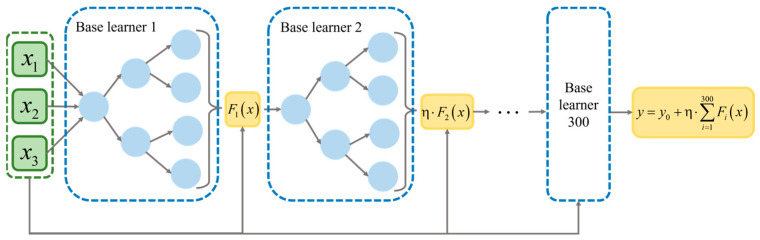
GBDT moder structure diagram.

To improve the accuracy of the algorithm, the parameters of the k-th decision tree are optimized by minimizing a loss function. In order to enhance the model’s fitting capability while keeping computational costs under control, the number of base learners is set to 300. To prevent both underfitting and overfitting, the learning rate is set to 0.1, and the maximum depth of each base learner is limited to 3. The overall structure of the model is illustrated in Figure 15.

### 5.3. SVM Model

SVM is an algorithm suitable for small sample, nonlinear, and high-dimensional data. The key function of this algorithm is to ensure that the learning machine has a certain learning ability, i.e., prediction function, learning function [43,44]. The basic type of SVM is

(16) $$\left\{\begin{array}{l} \min_{w,b}\frac{1}{2}\|w{\|}^{2}\\ y_{i}\left(w^{T}x_{i}+b\right)\geq 1,\;i=1,2,\ldots ,N \end{array}\right.$$

where w is the hyperplane normal vector, b is the function displacement term, $x_{i}$ represents the eigenvalue of the i data, and $y_{i}$ represents the label of the i data.

The core idea of SVM is to maximize the classification interval or minimize the prediction error by constructing the optimal hyperplane, so as to improve the generalization ability of the model. In this study, SVM is used to predict the roll surface temperature field. Considering the data characteristics and modeling requirements of this task, Gaussian Radial Basis Function (RBF) is selected as the kernel function. In order to avoid underfitting or overfitting of the model, the kernel width parameter gamma of RBF is set to 0.1, and the penalty parameter C is set to 10. The final regression function is

(17) $$f(x)=\sum_{i=1}^{n}\left(\alpha_{i}-\alpha_{i}^{*}\right)\exp\left(-\gamma {\left\|x_{i}-x\right\|}^{2}\right)+b$$

where $\alpha_{i}$, $\alpha_{i}^{*}$ are Lagrange multipliers, b is bias terms, and γ is hyperparameters of the Gaussian kernel.

### 5.4. Analysis of Model Results

The prediction model was established by taking the diameter, angle, and axial position of water spray hole as input values and the surface temperature of the roll as the output value. All the machine learning model training and prediction experiments were carried out on the Windows 10 platform, and the model program was realized by Python 3.8. All the machine learning model datasets were divided in the same proportion, with the training set accounting for 80% of the total sample size and the test set for 20%.

**Figure 16:**
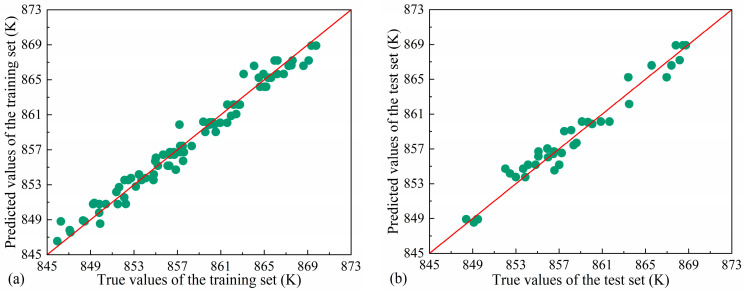

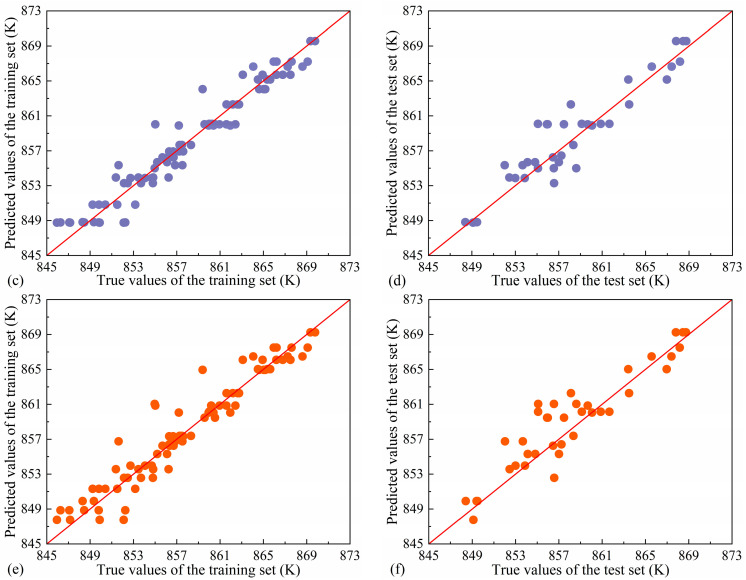
(**a**) RF model training set, (**b**) RF model test set, (**c**) GBDT model training set, (**d**) GBDT model test set, (**e**) SVM model training set, and (**f**) SVM model test set prediction results.

Figure 16 shows the prediction results of each model in the training set and test set. Overall, the RF model is more accurate than the GBDT model and SVM model, and its overall error control is better; the RF model has no large error prediction value in all data sets. In addition, the RF model performs well in the training set and test set, and there is no obvious overfitting phenomenon. In contrast, the fitting and generalization results of the GBDT and SVM models have large deviations. From the point of view of prediction error, the RF model shows better robustness and generalization ability in this prediction task.

In order to accurately analyze the accuracy of the model, the same evaluation indicators are used for all models in this study: mean square error MSE, mean absolute error MAE, coefficient of determination R^2^.

The formula of each evaluation index containing m samples is shown as follows:

Mean Square Error (MSE):

(18) $$\mathrm{MSE}=\frac{1}{m}{\sum_{i=1}^{m}\left(y_{i}-{\hat{y}}_{i}\right)}^{2}$$

Mean absolute error (MAE):

(19) $$\mathrm{MAE}=\frac{1}{m}\sum_{i=1}^{m}\left|y_{i}-{\hat{y}}_{i}\right|$$

Determination coefficient (R^2^):

(20) $$R^{2}=1-\frac{\sum {\left(y_{i}-{\hat{y}}_{i}\right)}^{2}}{\sum {\left({\bar{y}}_{i}-y_{i}\right)}^{2}}$$

where $y_{i}$ is the true value, ${\hat{y}}_{i}$ is the predicted value, and ${\bar{y}}_{i}$ is the average of the true values.

Table 4 shows the RMSE, MAE, and R^2^ values for each model on both the training and test sets. The RF model achieved the lowest RMSE (1.5266 and 1.7336) and MAE (1.1061 and 1.3203) values on both the training and test sets, while having the highest R^2^ (0.9387 and 0.9082), indicating that it not only has strong fitting ability but also has good generalization performance.

In summary, the RF model demonstrated the best overall performance in this study, while the GBDT and SVM models exhibited overfitting and underfitting, respectively, leading to relatively poorer prediction accuracy.

### 5.5. PSO Model

PSO was proposed by Eberhart and Kennedy in 1995. It is a global optimization method based on swarm intelligence [45]. In this algorithm, each particle represents a potential solution, moves continuously in the search space, and dynamically updates its position and velocity through its historical optimal position and the information of the optimal position in the population, so as to gradually approach the optimal solution of the problem

(21) $$\begin{array}{l} v_{i}^{sc}=\omega \cdot v_{i}^{sc}+c_{1}\;\mathrm{rand}\;(1)\cdot \left({\mathrm{Pbest}\;}^{sc}-x_{i}^{sc}\right)+c_{2}\;\mathrm{rand}\;(2)\cdot \left({\mathrm{Gbest}\;}^{sc}-x_{i}^{sc}\right)\\ x_{i}^{sc}=x_{i}^{sc}+0.5v_{i}^{sc} \end{array}$$

where ω is inertia weight; $c_{1}$ and $c_{2}$ affect convergence speed and global search ability, representing individual and group learning factor; and rand (1), rand (2) are random value functions that control the degree to which particles approach the optimal position, which increases the diversity of search [46].

Traditional fixed-parameter settings often struggle to balance global exploration and local exploitation when dealing with diverse optimization problems, thereby limiting the adaptability and robustness of the algorithm. To address this issue, an adaptive strategy is introduced in this study, enabling dynamic adjustment of parameter settings based on the iterative process of the algorithm. Specifically, a Particle Swarm Optimization (PSO) algorithm is integrated with the Random Forest (RF) model to optimize the geometric parameters of the spray ring. In this approach, each parameter combination is evaluated using cross-validation on the training set to determine the fitness value of each particle. Based on the individual best and global best positions during iterations, the optimal parameter combination is ultimately identified. To obtain the spray ring geometry that minimizes the roll surface temperature, PSO is further combined with response surface analysis. This requires the construction of a corresponding multi-objective optimization function subject to relevant constraints. Based on the input–output characteristics, the constraint ranges are defined as follows [47,48]:

(22) $$\begin{array}{l} \underset{h,\alpha ,d}{\min}\;\mathrm{Loss}\;(h,\alpha ,d)\\ s.t.\left\{\begin{array}{l} 20\leq h\leq 100\\ 0\leq \alpha \leq 24\\ 2\leq d\leq 4 \end{array}\right. \end{array}$$

where h is axial position, α is angle, and d is diameter.

Based on the overall optimization results, it can be concluded that employing the PSO algorithm to identify the optimal geometric parameter combination corresponding to the minimum roll surface temperature is both feasible and effective. The optimal parameter set obtained through this process is listed in Table 5.

**Figure 17:**
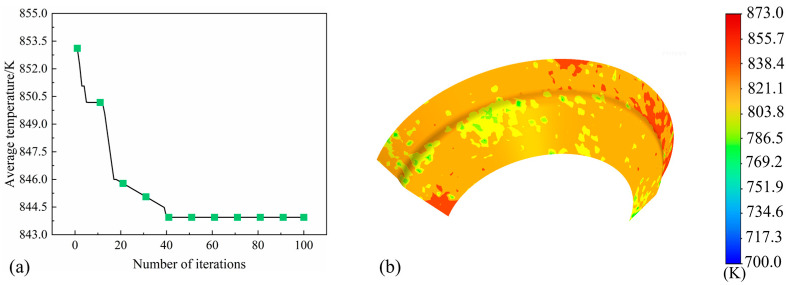
(**a**) Iterative result graph and (**b**) temperature cloud graph under the optimal parameters.

As shown in Figure 17a, the algorithm exhibits rapid convergence in the early stages, with convergence gradually stabilizing after approximately 40 iterations. The overall results demonstrate the feasibility of using the PSO algorithm to identify the optimal parameter combination that minimizes the average surface temperature of the roll. Based on the geometric parameters optimized by the PSO-RF model, a new simulation was conducted, and the resulting temperature contour map of the roll surface is presented in Figure 17b. Experiments were also carried out on the experimental bench, and the heat transfer coefficient was found to be 4710.5 W/m^2^·K. Compared with the parameters of the water spray ring used in the current three-roll planetary rotary roll cooling system (with a diameter of 2.5 mm, an angle of 18°, and an axial distance of 60 mm), the heat transfer coefficient increased by 44.72%, as shown in Table 6. These results indicate that the optimized parameter combination significantly improves the cooling effect on the roll surface temperature.

## 6. Conclusions

In this paper, the effect mechanism of geometric parameters and axial position of water spray ring on the distribution characteristics and homogenization effect of roll surface temperature field in a three-roll planetary rolling system is systematically investigated through the deep fusion of a multi-physical field coupling simulation and machine learning algorithm. The research results show the following:

1. The flow rate and volume of cooling liquid show a high degree of sensitivity to the diameter and angle parameters of the water spray holes. When the angle of the water spray holes increases from 0° to 24° and the diameter increases from 2 mm to 4 mm, both the flow rate and volume of cooling liquid show a significant downward trend. However, within the range of 18° to 24°, the decrease is reduced.
2. The roll surface temperature field is obviously affected by the geometric parameters and axial position of the spray ring, which shows that the average temperature in the cooling zone of the roll surface increases gradually, with the increase of the spray hole diameter from 2 mm to 4 mm, and the cooling effect decreases greatly. With the increase of the angle, the average value of the surface temperature field of the roll shows a fluctuating change of first decreasing and then increasing. With the increase of the diameter, the change point shifts from 18° to 6–12°. The axial position of spray ring has obvious influence on the temperature field and cooling effect of roll surface, and the effect of spray hole diameter and angle should be considered comprehensively. Within the range of 0° to 18°, the cooling effect improves with the increase of distance. However, at a 24° spray hole angle, the cooling effect deteriorates as the spray distance increases. However, this trend will be alleviated by the increase in the diameter of the spray holes.
3. Based on several machine learning algorithms, the roll surface temperature under different parameters of spray ring was predicted, among which the RF model had the best fitting and generalization effect, GBDT was relatively inferior, and SVM had the worst effect. For the test set, the RMSE, MAE, and R^2^ of the RF model test set are 1.7336, 1.3203, and 0.9082, respectively.
4. The geometric parameters of the spray ring were optimized using the PSO algorithm in combination with the RF model. The lowest overall roll surface temperature was achieved when the axial distance of the spray ring was 96 mm, the spray angle was 7°, and the nozzle diameter was 2 mm. Under these conditions, the heat transfer coefficient was 4710.5 W/m^2^·K, which was 44.72% higher than that obtained with the actual parameters.

## Figures and Tables

**Table 1:** Simulation process parameters.

	Water Spray Ring Model		Fluid-Solid-Heat Coupling Model		
	Blowhole Diameter (d)/mm	Spray Angle (*α*)/°	Blowhole Diameter (d)/mm	Spray Angle (*α*)/°	Axial Position (h)/mm
Value	2	0	2	0	20
	2.5	6	2.5	6	40
	3	12	3	12	60
	3.5	18	3.5	18	80
	4	24	4	24	100

**Table 2:** Thermophysical and simulation properties of material.

Characteristic Parameter	Rolls	Cooling Liquid
Density kg/m^3^	7800	989.65
Specific Heat J/(kg∙K)	460	4089.6
Thermal Conductivity W/(m∙K)	26.14	0.5765
Dynamic Viscosity Pa·s		0.00113

**Table 3:** Experimental measurement temperature.

Axial Position/mm	Experimental Results/K	Simulation Results/K
20	393.9	854.7
40	391	853.9
60	387.3	852.7
80	390.2	853

**Table 4:** Evaluation index of each model.

Model	Training Set			Test Set		
	RMSE	MAE	R^2^	RMSE	MAE	R^2^
RF	1.5266	1.1061	0.9387	1.7336	1.3203	0.9082
GBDT	1.5532	1.1112	0.9361	2.0020	1.5352	0.8776
SVM	1.7511	1.1304	0.9190	2.3638	1.8094	0.8293

**Table 5:** Optimal parameter combination.

Parameter Variable	Optimal Solution
*h*/mm	96
α/°	7
*d*/mm	2

**Table 6:** Comparison of results before and after optimization.

*h*/mm	α/°	*d*/mm	Average Temperature/K	Heat Transfer Coefficient/(W/m^2^·K)
60	18	2.5	387.3	3254.7
96	7	2	341.5	4710.5

## Data Availability

The original contributions presented in this study are included in the article. Further inquiries can be directed to the corresponding author.
